# The Effects of Cold Acclimation on Cold Tolerance and Growth and Reproduction of *Plodia interpunctella*

**DOI:** 10.3390/insects16090927

**Published:** 2025-09-03

**Authors:** Zhuoke Shi, Huiyuan Zhang, Shaohua Lu, Mingshun Chen

**Affiliations:** 1 School of Food and Strategic Reserves, Henan University of Technology, Zhengzhou 450001, China; shizhuokeyx@163.com (Z.S.); huiyuanzhangvip@163.com (H.Z.); 2 Department of Entomology, Kansas State University, Manhattan, KS 66506, USA; mchen@ksu.edu

**Keywords:** *Plodia interpunctella*, cold acclimation, supercooling point, cold tolerance, stored grain pest

## Abstract

*Plodia interpunctella* is a globally significant pest of stored grains, posing a major threat to food safety. To explore its cold-adaptation mechanisms, this study evaluated the physiological and developmental responses of different life stages following short-term cold acclimation at 4 °C. Results showed that cold acclimation significantly reduced the supercooling points (SCPs) of larvae and pupae, with the greatest reduction observed in the second instar larvae. Antioxidant enzyme assays revealed marked increases in the activities of superoxide dismutase (SOD), catalase (CAT), and peroxidase (POD), indicating enhanced oxidative stress resistance. Developmental durations were significantly shortened at lower temperatures in acclimated individuals, and fecundity was notably increased at 24 °C, although no significant changes were observed at higher temperatures. These findings suggest that cold acclimation improves the cold tolerance and reproductive performance of *P. interpunctella* under low-temperature conditions, offering insights into insect adaptability and providing theoretical support for the development of low-temperature-based pest management strategies in stored grain systems.

## Simple Summary

This study investigates the effects of cold acclimation on the cold tolerance, development, and reproduction of the *Plodia interpunctella*. The results show that cold acclimation primarily enhances the cold tolerance of *P. interpunctella* by lowering its supercooling point (SCP) and activating the antioxidant enzyme system. Additionally, cold acclimation also regulates the oviposition behavior of adults and accelerates their developmental process under low-temperature conditions. These findings provide potential applications for the green pest control strategies of grain storage pests and contribute to the development of precise pest management measures under various ecological and climatic conditions.

## 1. Introduction

Food is a fundamental resource underpinning public well-being. The efficiency of its storage and utilization directly influences social development and food safety [1,2]. Although recent advances in agricultural productivity and postharvest technologies have led to increasingly sophisticated grain storage systems, postharvest losses during storage remain a critical challenge. According to the Food and Agriculture Organization (FAO), approximately 14% of global food production is lost annually due to inadequate storage, transportation, and processing infrastructure [3,4,5]. In China, although on-farm grain losses have been reduced from about 8% to around 3% due to improved storage technologies and infrastructure upgrades, pest-related losses still amount to the equivalent of millions of hectares of grain output, owing to the large storage volume and wide variability in storage conditions [6,7]. Consequently, enhancing grain storage management and promoting technological innovation are vital to ensuring food safety.

*Plodia interpunctella* (Hübner, 1813), commonly known as the Indian meal moth (Lepidoptera: Pyralidae), is a cosmopolitan and economically important pest of stored products [8,9]. It reproduces rapidly and has a broad food range, feeding on various substrates such as stored grains, agricultural by-products, herbal medicines, dried fruits, preserved foods, and animal hides [3,10]. Larval infestation is destructive, as larvae spin silk webs that bind feeding substratum into clumps, localizing heat and humidity. These conditions often result in surface condensation, mold growth, and ultimately the spoilage of stored products [11]. Currently, chemical fumigation and low-oxygen controlled atmospheres are the primary methods used to manage storage pests. Among these, phosphine (PH_3_) fumigation remains widely adopted and effective [12,13]. However, the overuse of chemical agents has led to the emergence of the “3R” problem: pest resistance, chemical residues, and pest resurgence [14,15]. While low-oxygen treatments eliminate pesticide contamination, they require highly airtight storage, involve prolonged treatment durations, risk reinfestation after reoxygenation, and may impair seed viability and accelerate grain degradation [16]. Therefore, the development of sustainable, eco-friendly pest control strategies is urgently needed.

Low-temperature grain storage has emerged as an effective method to suppress insect development by reducing metabolic rates [17]. As poikilothermic organisms, insects are highly sensitive to ambient temperature, which directly governs their development, reproduction, and overall life history [18,19]. Low temperatures significantly slow insect growth and reproduction, and subcritical temperatures may induce diapause, thereby enhancing environmental resilience [20,21,22,23]. Near or below 0 °C, insects may enter chill coma or die due to severe metabolic suppression [24]. For instance, cold acclimation has been shown to prolong development and reduce fecundity in *Halyomorpha halys* and *Bactrocera* spp. [25,26]. Cold acclimation is a physiological process that enhances insect tolerance to low temperatures by adjusting metabolic and biochemical pathways [27,28,29,30]. For example, cold-acclimated *Locusta migratoria* adults exhibit faster chill coma recovery, and *Belgica antarctica* larvae show higher survival rates after freezing when pre-exposed to sublethal cold [29,30]. These adaptations often involve the expression of cold-resistance genes, modulation of supercooling point (SCP), and enhancement of antioxidant defense systems [31]. Reactive oxygen species (ROS), which accumulate under stress, can damage cellular components such as lipids, proteins, and nucleic acids [32,33]. To mitigate this oxidative damage, insects activate antioxidant enzymes including superoxide dismutase (SOD), catalase (CAT), and peroxidase (POD) [34,35]. Studies have shown that cold acclimation increases the activity of these enzymes, thereby enhancing resistance to oxidative stress and improving survival in cold environments [36,37,38].

Although low-temperature storage effectively limits pest development and reproduction, cold acclimation may inadvertently enhance an insect’s cold tolerance over time, posing new challenges for pest control [39,40]. Considerable progress has been made in elucidating the physiological mechanisms underlying cold acclimation in insects, particularly with respect to survival, development, and stress-response pathways [41,42]. However, most of this knowledge derives from studies on non-storage insects, whereas investigations focusing on stored-product pests remain relatively limited. In the case of *P. interpunctella*, previous studies have primarily focused on general survival and developmental responses to cold acclimation, while the involvement of antioxidant defense systems and potential effects on reproductive capacity are still poorly understood. Adler [43] examined the survival and developmental responses of *P. interpunctella*, under low-temperature conditions, demonstrating the potential utility of cold treatment as a control strategy. Closing these knowledge gaps is essential for evaluating whether cold acclimation diminishes the long-term effectiveness of low-temperature storage as a sustainable pest management approach.

Therefore, in this study, *P. interpunctella* was used as a model species to assess the effects of cold acclimation at 4 °C on supercooling point, antioxidant enzyme activity, developmental duration across various life stages, and reproductive capacity. By elucidating the physiological mechanisms underlying cold tolerance, this research provides a theoretical foundation for developing precise and environmentally friendly pest management practices under low-temperature storage conditions.

## 2. Materials and Methods

### 2.1. Insects

The *P. interpunctella* population used in this study originated from a long-term laboratory colony maintained at the Stored Product Insect Research Laboratory, Henan University of Technology. The insects had been continuously reared for more than 10 generations on a standard artificial diet composed of rolled oats and yeast powder at a mass ratio of 19:1. Rearing conditions were maintained in a climate-controlled incubator set at 30 ± 2 °C, 70 ± 5% relative humidity, and a 16L:8D h photoperiod.

### 2.2. Determination of Larval Body Coloration After Cold Acclimation

The 2nd instar and 4th instar larvae of *P. interpunctella* (30 individuals per instar, with an equal number of males and females) were randomly selected and observed under a microscope. The sex of each larva was determined based on morphological characteristics at the posterior end of the abdomen. Males were distinguished by the presence of a dark patch on the abdomen, which becomes visible in the 4th instar. To ensure accurate sexing, the 2nd instar larvae were continuously reared until adult emergence, at which point their sex was verified. The larvae were divided into two groups: a control group, which received no cold acclimation and was maintained at room temperature, and a treatment group, which underwent cold acclimation at 4 °C for 15 h. After acclimation, they were immediately removed and their body coloration was observed under a super-depth microscope (Model VHX-5000, Keyence Corporation, Osaka, Japan).

### 2.3. Measurement of Supercooling Point of Cold Acclimated P. interpunctella

Individuals of the *P. interpunctella* at different developmental stages (2nd instar larvae, 4th instar larvae, pupae, and adults) were randomly selected (30 individuals per stage) and subjected to either a control group or a cold-acclimated treatment group. The treatment group was subjected to cold acclimation at 4 °C for 15 h in a temperature-controlled freezer (Model BC-153KM, Midea Group, Foshan, Guangdong, China), while the control group remained at room temperature without acclimation. After treatment, the insects were removed and allowed to recover room temperature (30 ± 2 °C) for less than 1 h before subsequent measurements. For each measurement, a single insect was placed in a 0.5 mL PCR tube, with a thermistor probe placed in close contact with the insect’s body surface. The tubes were then transferred to a −25 °C temperature-controlled freezer (Model BC-153KM, Midea Group, Foshan, Guangdong, China). Body temperature was continuously monitored using a computerized supercooling point detection system (Model SUN-V, Beijing Pengcheng Electronic Technology Center, Beijing, China) and associated software, recording data at a frequency of one reading per second. The supercooling point (SCP) was determined from the temperature curve generated during the cooling process.

### 2.4. Determination of Antioxidant Enzymes Activity of Cold Acclimated P. interpunctella

Individuals of *P. interpunctella* at different developmental stages (2nd instar larvae, 4th instar larvae, pupae, and adults) were randomly assigned to two groups: a control group (cold acclimation at 4 °C for 0 h) and a treatment group (cold acclimation at 4 °C for 15 h). For each biological replicate, 30 whole insects (from the same developmental stage and treatment group) were collected and pooled. From these pooled whole-insect homogenates, approximately 100 mg of the coarse-ground material was carefully weighed and transferred into a 2 mL pre-chilled centrifuge tube containing phosphate-buffered saline (PBS, pH 7.4, pre-cooled to 4 °C) and two stainless steel grinding beads. The tissues were homogenized using a tissue grinder and centrifuged at 3000 rpm for 20 min. The supernatant was collected and stored at −20 °C for subsequent analysis. The activities of the three test enzymes were determined using insect enzyme immunoassay kits (ml036253 [SOD], ml062687 [CAT], and ml062688 [POD]), respectively (Shanghai Enzyme-linked Biotechnology Co., Ltd., Shanghai, China). A double-antibody sandwich protocol was employed: microplates were coated with capture antibodies, followed by sequential addition of samples and HRP-conjugated detection antibodies to form an antibody–antigen–enzyme complex. After sterile washing, 3,3′,5,5′-Tetramethylbenzidine (TMB) substrate was added, and color development was measured at 450 nm using a microplate reader. Enzyme activities were calculated using standard curves. Each treatment included three biological replicates, and each sample was tested in triplicate.

### 2.5. Development, Lifespan and Fecundity of Acclimated P. interpunctella Depending on Temperature

Fifty newly laid (1-day-old) *P. interpunctella* eggs were placed in 15 mm-diameter polypropylene cups. Each cup contained 50 eggs, which were assigned to different treatment groups. Control groups were reared at room temperature, while treatment groups were acclimated at 4 °C for 15 h. After treatment, the samples were transferred to incubators (SPX-250, Beijing Yongguangming Medical Instrument Factory, Beijing, China) set at three temperature regimes: 24 °C, 28 °C, and 32 °C (±1 °C), under a 16L:8D h photoperiod. The insect colony was maintained at these temperatures throughout the entire developmental cycle (egg, larva, pupa, and adult). For fecundity assessment, newly emerged males and females were paired in custom-made oviposition containers consisting of two 15 mm-diameter polypropylene cups separated by a 40-mesh nylon layer, allowing oviposition while preventing escape. After confirmed mating, individual females were transferred to separate oviposition units for continuous monitoring. The number of eggs laid was recorded daily until the death of the female, and total lifetime fecundity were calculated. Developmental durations of each life stage and total lifespan were recorded.

### 2.6. Data Analysis

All statistical analyses were conducted using the SPSS software (version 26, IBM, Inc., Armonk, NY, USA). Data were first tested for normality and homogeneity of variance. For data that did not meet the assumptions of normal distribution, an arcsine square root transformation was applied. One-way analysis of variance (ANOVA) was used to evaluate differences in supercooling point (SCP) among developmental stages as well as developmental duration and fecundity across different temperature treatments. Independent samples t-tests were performed to compare the indices between cold-acclimated and non-acclimated groups within the same developmental stage. When significant differences were detected, Duncan’s multiple range test was used for post hoc comparisons. Statistical significance was considered at *p* < 0.05.

## 3. Results

### 3.1. Effects of Cold Acclimation on Body Coloration

**Figure 1:**
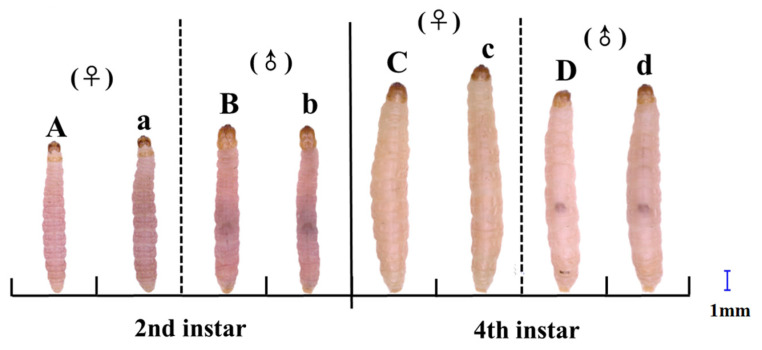
Morphological changes in body pigmentation of the second and fourth instars *P. interpunctella* larvae following cold acclimation treatment. Uppercase letters in the figure represent larvae before cold acclimation, and lowercase letters denote larvae after cold acclimation.

After cold acclimation, the body coloration of *P. interpunctella* larvae became darker (Figure 1). Under control conditions, the second instar larvae exhibited a pale pink hue (Figure 1A,B), while the fourth instar larvae appeared pale yellow to off-white (Figure 1C,D). In contrast, after 15 h of cold acclimation at 4 °C, larvae at both instars showed deeper pigmentation, with body color shifting toward purple or gray tones (Figure 1a–d). These observations suggest that cold acclimation has an effect on larval pigmentation in *P. interpunctella*.

### 3.2. Effects of Cold Acclimation on Supercooling Point

**Figure 2:**
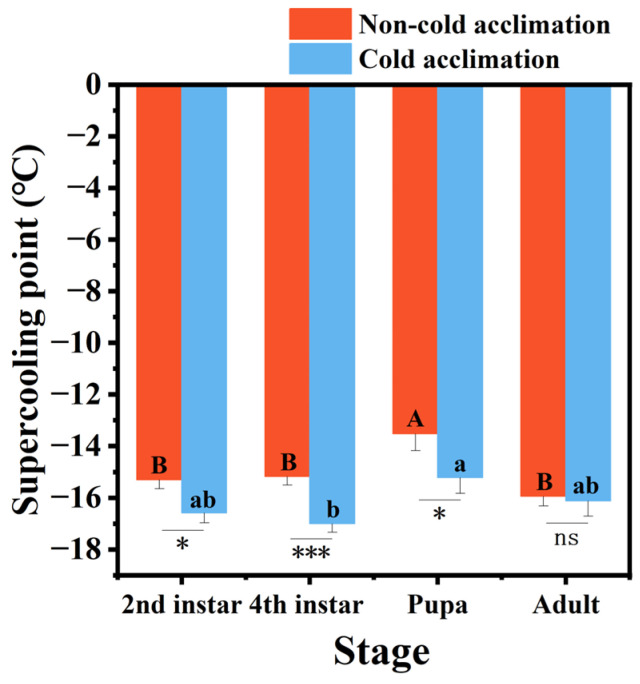
Supercooling point of *P. interpunctella* at different developmental stages under cold-acclimated and non-acclimated conditions. Supercooling point of *P. interpunctella* at different developmental stages under cold-acclimated and non-acclimated conditions. Data represent mean ± standard error. Different uppercase letters indicate significant differences in supercooling point among developmental stages before cold acclimation, while different lowercase letters indicate significant differences after cold acclimation. The asterisk denotes a statistically significant difference in supercooling point (SCP) between cold-acclimated and control groups of *P. interpunctella*. ns means no significant difference, * means *p* < 0.05, and *** means *p* < 0.001.

Cold acclimation had no significant effect on the supercooling point (SCP) of *P. interpunctella* adults, but it significantly decreased the SCPs of the second instar larvae, fourth instar larvae, and pupae (Figure 2). Prior to acclimation, the SCPs of larvae and adults were significantly lower than that of the pupae. After cold acclimation, the SCPs of the second instar larvae, fourth instar larvae, and pupae decreased by 1.29 °C, 2.82 °C, and 2.10 °C, respectively. Notably, the SCP of the fourth instar larvae became significantly lower than that of the pupae. These results indicate that cold acclimation significantly improved cold tolerance in the larval stages of *P. interpunctella*, particularly in the fourth instar larvae, which showed the greatest sensitivity to cold acclimation. In contrast, adults exhibited the weakest cold stress adaptation to acclimation.

### 3.3. Effects of Cold Acclimation on Antioxidant Enzyme Activities

**Figure 3:**
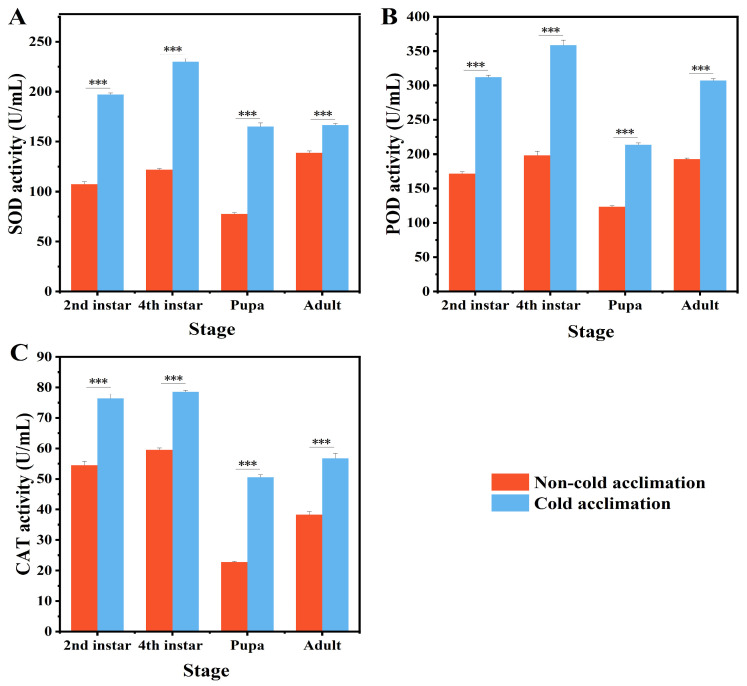
Changes in the activities of SOD (**A**), POD (**B**), and CAT (**C**) in *P. interpunctella* under cold-acclimated and non-acclimated conditions. SOD: superoxide dismutase, CAT: catalase, POD: peroxidase. Data represent mean ± SEM. Asterisks indicate significant differences between control and treatments. *** means *p* < 0.001.

Cold acclimation significantly altered antioxidant enzyme activities across all developmental stages of *P. interpunctella* (Figure 3). Superoxide dismutase (SOD) activity was significantly increased following acclimation (*p* < 0.001) (Figure 3A). All life stages showed a positive response, though the magnitude of increase varied. In the fourth instar larvae, SOD activity nearly doubled from 121.88 ± 1.18 U/mL to 229.88 ± 3.25 U/mL. In contrast, adult SOD activity showed a modest increase, from 138.65 ± 1.82 U/mL to 166.51 ± 1.55 U/mL. Peroxidase (POD) activity also increased significantly in all stages (*p* < 0.001), with the highest increase in the fourth instar larvae and the lowest in pupae (Figure 3B). Catalase (CAT) activity followed a similar trend. The most substantial increase was observed in pupae, where activity rose from 22.75 ± 0.18 U/mL to 50.51 ± 0.82 U/mL (Figure 3C). The increase in larvae was comparatively smaller but still statistically significant.

### 3.4. Effects of Cold Acclimation on Development, Lifespan and Reproduction

**Figure 4:**
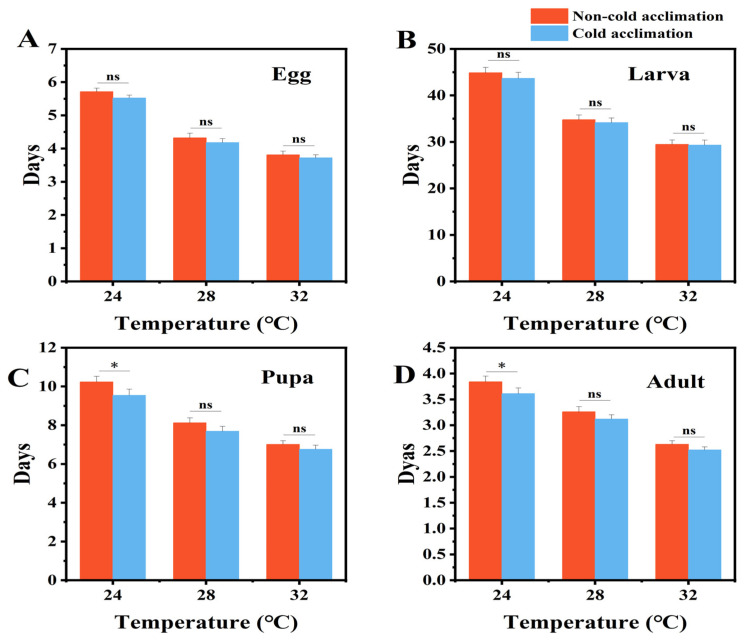
Developmental and survival period of *P. interpunctella* egg (**A**), larva (**B**), pupa (**C**), and adult (**D**) under cold-acclimated and non-acclimated conditions. Under three temperature regimes (24 °C, 28 °C, and 32 °C). Data represent mean ± SEM. Asterisks indicate significant differences between control and treatments. ns means no significant difference, and * means *p* < 0.05.

Cold acclimation affected the developmental duration of *P. interpunctella* at different temperatures (Figure 4). At 24 °C, the developmental period of eggs was reduced from 5.71 ± 0.11 days to 5.52 ± 0.09 days after acclimation (Figure 4A), although the difference was not statistically significant. A similar trend was observed in the larval stage, with mean larval development duration decreasing from 44.86 ± 1.15 days in the control group to 43.65 ± 1.32 days in the cold-acclimated group (Figure 4B). However, significant reductions were found in the pupal and adult stages at 24 °C (*p* < 0.05) (Figure 4C,D). The pupal stage shortened from 10.23 ± 0.29 to 9.54 ± 0.32 days, and the adult stage from 3.84 ± 0.11 to 3.61 ± 0.11 days. Overall, the total lifespan of cold-acclimated individuals was significantly shorter at lower temperatures, exactly 24 °C (sum of 62.32 ± 1.84 days compared to the 64.64 ± 1.66 days registered for the control), while no significant differences were observed at 28 °C (sum of 49.15 ± 1.43 days versus the 50.46 ± 1.54 days) or 32 °C (sum of 42.32 ± 1.42 days compared to the 42.9 ± 1.35 days for the control).

**Figure 5:**
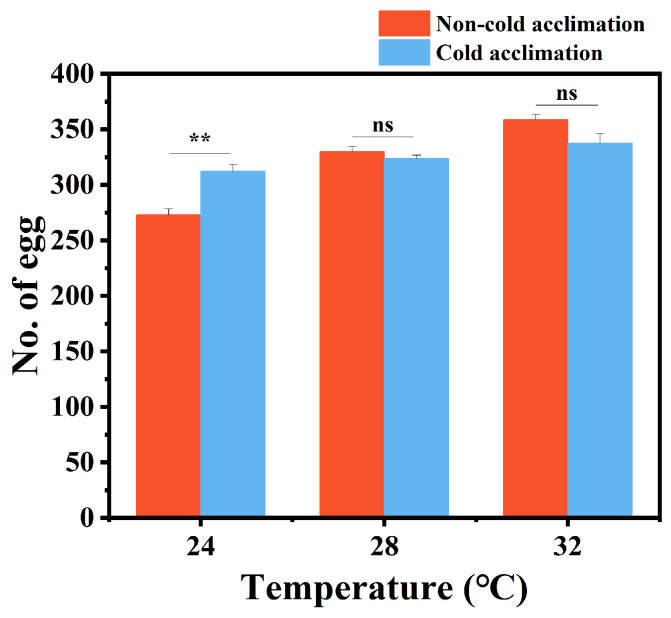
Mean egg production of *P. interpunctella* females under cold-acclimated and non-acclimated conditions at different temperatures. Data represent mean ± SEM. Asterisks indicate significant differences between control and treatments. ns means no significant difference, and ** means *p* < 0.01.

Cold acclimation also influenced oviposition (Figure 5). At 24 °C, the average number of eggs laid per female increased significantly from 273 to 312 (*p* < 0.01) after acclimation. However, at 32 °C, the number of eggs decreased slightly from 359 to 338, though the difference was not significant (*p* > 0.05). No significant changes were observed at 28 °C. These results suggest that cold acclimation enhances reproductive performance at lower temperatures but has limited impact under warmer conditions.

## 4. Discussion

This study demonstrates that short-term cold acclimation induces a suite of physiological, phenotypic, and reproductive changes in *P. interpunctella*, collectively enhancing its capacity to survive and function under low-temperature conditions. After exposing individuals at different developmental stages to 4 °C for 15 h and subsequently 1 h at 30 °C, we observed significant improvements in cold tolerance, as evidenced by reduced supercooling points (SCPs), enhanced antioxidant enzyme activity, darkened body coloration, accelerated development, and increased oviposition at 24 °C. These findings highlight the complex and stage-specific nature of cold acclimation responses and underscore their potential significance for pest management in low-temperature storage environments.

Phenotypic plasticity in insect pigmentation is often influenced by environmental temperature, with cooler conditions inducing darker coloration in many species [44]. In our study, the larvae of *P. interpunctella* developed darker body coloration following cold acclimation (Figure 1). This observation is consistent with previous studies in *Spodoptera exempta* [45] and *Drosophila melanogaster* [46], which propose that darker pigmentation facilitates heat absorption and retention, improving thermoregulation in poikilotherms [47].

As poikilotherms, insects frequently rely on lowering their SCP to survive subzero conditions [48,49,50]. Our results showed significant decrease in SCPs for larvae and pupae following cold acclimation, particularly in the fourth instar larvae. However, cold acclimation had little effect on adult SCPs and failed to significantly improve cold tolerance in pupae and adults. This phenomenon may reflect differences in physiological states and plasticity across developmental stages. Larvae are generally more metabolically active and capable of rapidly initiating physiological responses such as the synthesis of cryoprotectants and ion regulation [51]. In contrast, the pupal stage is primarily focused on tissue reorganization, which may limit its capacity for physiological adjustment [52]. Adults, having completed development, may also exhibit reduced flexibility in responding to environmental stress [53]. Consistently with this pattern, previous studies on *P. interpunctella* have shown that cold acclimation significantly lowers larval SCPs, especially in later instars, but has limited effects on pupae and adults, thereby underscoring stage-specific physiological plasticity [54]. Comparable findings have been reported for *Phthorimaea operculella,* where short-term cold acclimation markedly lowered SCPs in larvae but left pupae largely unaffected, further confirming that larval stages exhibit higher physiological flexibility in response to low-temperature stress [55]. Therefore, the ability of *P. interpunctella* to respond to cold acclimation may be largely influenced by the physiological characteristics specific to each developmental stage. This suggests that SCP decreasing alone is not sufficient to fully explain cold resistance in later stages, which may require activation of additional pathways such as antifreeze protein synthesis, cryoprotectant accumulation (e.g., glycerol, trehalose), or adenosine monophosphate-activated protein kinase (AMPK)-mediated metabolic regulation [56,57,58].

Cold acclimation often induces the overproduction of reactive oxygen species (ROS), which can damage cellular macromolecules [59,60,61]. In response, insects activate enzymatic antioxidants such as SOD, CAT, and POD to mitigate oxidative damage and restore redox balance [62]. In the current study, cold acclimation significantly upregulated SOD, CAT, and POD activities across all developmental stages, especially in larvae and pupae (Figure 3). SOD and POD activity nearly doubled in the fourth instar larvae, while CAT activity showed the largest increase in pupae, suggesting stage-specific antioxidant responses. Although cold acclimation did not significantly change the SCP in adults, the observed increase in antioxidant enzyme activity suggests that physiological responses may still occur at the biochemical level, indicating a stage-specific cold adaptation strategy. These enzymatic responses may be part of a broader stress-response pathway regulated by nuclear factor erythroid 2-related factor 2 (Nrf2)-like transcription factors, as demonstrated in *Drosophila suzukii* [63]. Additionally, cryoprotectants accumulated during acclimation, such as glycerol or sorbitol, may stabilize cellular membranes, buffer ROS levels, and reduce lipid peroxidation through enzyme–membrane interactions [64,65]. These results support the hypothesis that cold acclimation enhances oxidative stress tolerance, which plays a vital role in an insect’s cold hardiness [66].

Beyond stress resistance, cold acclimation significantly impacted the life history traits of *P. interpunctella*. At 24 °C, cold-acclimated individuals exhibited shortened developmental durations, especially during the pupal stage, but shortened lifespan in adults (Figure 4). This may reflect improved energy utilization or enhanced metabolic enzyme function under mild cold conditions. Moreover, cold acclimation increased female fecundity at 24 °C (Figure 5), while no significant changes were observed at 28 °C or 32 °C, suggesting that the reproductive benefits of acclimation are temperature dependent. In addition, cold acclimation has been shown to alter the developmental timing of *P. interpunctella*, indicating that acclimation can influence both growth rate and reproductive output [67]. Similar temperature–acclimation interactions have also been reported in *Drosophila melanogaster*, where pre-exposure to cooler conditions accelerated development under suboptimal thermal environments [68]. These observations indicate that acclimation history plays a critical role in shaping developmental and reproductive strategies. It is important to consider that temperature is not the sole factor influencing insect performance. Environmental variables such as humidity also modulate developmental and reproductive outcomes [68,69]. For example, the development of *Drosophila suzukii* is jointly affected by relative humidity and temperature [69]. Future work should examine how these factors interact with cold acclimation to determine *P. interpunctella*’s adaptive capacity under realistic storage conditions. 

## 5. Conclusions

Cold acclimation activates an integrated suite of responses—spanning morphological, physiological, and reproductive dimensions—that enable *P. interpunctella* to better tolerate and exploit cold environments. These findings contribute to a deeper understanding of insect cold adaptation and provide theoretical support for low-temperature-based pest management strategies. However, our experiments were conducted under controlled laboratory conditions. Future studies should incorporate dynamic environmental regimes (e.g., seasonal and diurnal temperature fluctuations) and test *P. interpunctella* populations from wild sources to better simulate real-world storage conditions and validate the practical relevance of acclimation-induced traits.

## Data Availability

The original contributions presented in this study are included in the article. Further inquiries can be directed to the corresponding author.
